# An Innovative Community‐led Education and Recruitment Program to increase Clinical Research Participation Among Black Americans

**DOI:** 10.1002/alz.090139

**Published:** 2025-01-09

**Authors:** Bahar Niknejad, Ethlyn McQueen Gibson, Travonia Brown‐Hughes, Katie McDonough, Ebony Andrews, Deborah Hudson, Hamid Okhravi

**Affiliations:** ^1^ Eastern Virginia Medical School, Norfolk, VA USA; ^2^ Norfolk State University, Norfolk, VA USA; ^3^ North Carolina Agricultural and Technical State University, Greensboro, NC USA; ^4^ Alzheimer’s Association, Norfolk, VA USA; ^5^ Hampton University, Hampton, VA USA

## Abstract

**Background:**

Previous studies attest to a lack of awareness about Alzheimer’s Disease (AD) and limited participation of Black Americans in AD clinical trials. The AHEAD Study is a multicenter trial focused on preventing AD by evaluating the effectiveness and safety of Lecanemab in individuals with preclinical AD. The study aims to recruit at least 15% from underrepresented populations, including Black Americans. To achieve this goal, our study site utilizes a community‐based participatory research (CBPR) approach and developed a Community Education and Recruitment Program (CERP) designed to increase the participation of Black individuals in the AHEAD Study.

**Method:**

In a collaborative partnership between researchers at Eastern Virginia Medical School, Hampton University, a Historically Black College University (HBCU) and the Alzheimer’s Association of Southeastern Virginia, a Community Advisory Board and Ministerial Alliance, comprised of Black community leaders in Hampton Roads, Virginia was formed. The board has directed the development of our CERP. Two Black community health workers (CHW) were trained using an educational toolkit centered on increasing AD awareness and community recruitment for the AHEAD Study. Within our CERP, our team, comprised of faculties, Alzheimer’s Association trained volunteers and CHWs, conducted educational programs at various Black community venues, including senior centers, health fairs, civics events, fraternities/sororities, and church events like Purple Sunday programs. Study outcomes metrics include: attendance at outreach events, the number of attendees who signed up for the study, the number of participants prescreened, and the number of participants randomized into the AHEAD Study.

**Result:**

As of January 2024, we have presented our program at 63 community events with over 6,300 attendees. We have held Purple Sunday luncheons and talks at over 18 Black churches in urban and rural localities in Hampton Roads. A total of 334 participants have signed up for the study, 180 have been prescreened, and 101 have passed the first phase of screening and 6 have been randomized in the AHEAD Study.

**Conclusion:**

Partnering with key community stakeholders thru a community‐led education and recruitment program has proven to be highly effective in circumventing sociocultural barriers to research participation by members of underrepresented groups.

